# Supplementary material for: Where are patients missed in the tuberculosis diagnostic cascade? A prospective cohort study in Ghana
Source: PLoS One. 2020 Mar 19;15(3):e0230604. doi: 10.1371/journal.pone.0230604 (PMC7081980; doi:10.1371/journal.pone.0230604)
Supplement: Attachment — Submitted filename: PONE-D-19-34140_reviewer.docx [file pone.0230604.s001.docx]

**Comments to the authors:**

The current study entitled “Where are patients missed in the tuberculosis diagnostic cascade? A prospective cohort study in Ghana” by Joyce B. Der et al. reported the possible reasons and to circumvents these in lower TB diagnosis rate in Ghana. They showed in the current study that distance of the diagnostic centers from the reporting center is the main hurdle to submit the sample for diagnosis. Although, the population study size chosen is moderate as well as the age and gender is somewhat biased. These could be managed at the time of patient recruitment. The study well is designed, executed and presented. Data are presented in legible manner and are easily understandable. Manuscript is well written. The authors tried to find the reasons behind the less reporting of the TB cases in a region of Ghana. They determined the casual link and suggested the measures to combat the problems. The information provided in the manuscript can be used to fill the gap that exist in the regions of Ghana and if followed may increase the cases of TB reporting and their diagnosis. These information’s can also be used to increase the rate of TB diagnosis and its management in high TB burden areas of the Ghana. The manuscript is suitable and could be considered for publication in PLOS ONE.
