# Supplementary material for: Where are patients missed in the tuberculosis diagnostic cascade? A prospective cohort study in Ghana
Source: PLoS One. 2020 Mar 19;15(3):e0230604. doi: 10.1371/journal.pone.0230604 (PMC7081980; doi:10.1371/journal.pone.0230604)
Supplement: Attachment — Submitted filename: Response to Reviewers.docx [file pone.0230604.s002.docx]

15^th^ February 2020

HASNAIN SEYED EHTESHAM
Academic Editor

PLOS ONE

Dear Prof Hasnain Seyed Ehtesham,

**PONE-D-19-34140: Where are patients missed in the tuberculosis diagnostic cascade?  A prospective cohort study in Ghana: Der J et al**

We thank the reviewers for their positive feedback and comments. The reviewers' comments are reproduced below in italics, followed by our responses in plain text. Sentences that have been added to the main manuscript are in font colour red.

***Reviewers’ comments:***

*Reviewer #1*

*Recording of positive family history of TB is not mentioned. if that is available in the records that should be mentioned.*

Thank you for this comment. Unfortunately we did not collect information on positive family history of TB and therefore cannot include it.

*Also if TB was suspected as a complication of HIV has not been recorded although presence / absence of HIV has been noted*

Thank you for the comment. We recruited people who were attending the general outpatients’ department using a referral system through the task shifting officer who is responsible for screening people with symptoms of TB and referring them to the laboratory for a sputum test. People attending for HIV care who needed investigation for TB were referred through this system and some will have been included in our study. We asked participants to report their HIV status if they knew it, and as you say we have reported this. However we did not ask participants their route of referral and so we are unable to report how many participants were referred from the antiretroviral (ART) clinic to the task shifting officer.

*Reviewer #2*

*The current study entitled “Where are patients missed in the tuberculosis diagnostic cascade? A prospective cohort study in Ghana” by Joyce B. Der et al. reported the possible reasons and to circumvents these in lower TB diagnosis rate in Ghana. They showed in the current study that distance of the diagnostic centers from the reporting center is the main hurdle to submit the sample for diagnosis. Although, the population study size chosen is moderate as well as the age and gender is somewhat biased. These could be managed at the time of patient recruitment. The study well is*

*designed, executed and presented. Data are presented in legible manner and are easily understandable. Manuscript is well written. The authors tried to find the reasons behind the less reporting of the TB cases in a region of Ghana. They determined the casual link and suggested the measures to combat the problems. The information provided in the manuscript can be used to fill the gap that exist in the regions of Ghana and if followed may increase the cases of TB reporting and their diagnosis. These information’s can also be used to increase the rate of TB diagnosis and its management in high TB burden areas of the Ghana. The manuscript is suitable and could be considered for publication in PLOS ONE.*

Thank you for the positive feedback about our study. In relation to the comment on age and sex being somewhat biased, our study was designed to consecutively select patients with symptoms of TB who have been routinely asked by a health worker to do a sputum test. We believe the study participants were therefore representative of people being asked to give a sputum for a TB test. We respectfully disagree with the comment. We have not changed the manuscript.

***Editorial points to be addressed:***

*Please ensure that your manuscript meets PLOS ONE's style requirements, including those for file naming. The PLOS ONE style templates can be found at*
[*http://www.journals.plos.org/plosone/s/file?id=wjVg/PLOSOne_formatting_sample_main_body.pdf*](https://protect-eu.mimecast.com/s/MYq-Ck714TnyloH9FFXk?domain=journals.plos.org) *and* [*http://www.journals.plos.org/plosone/s/file?id=ba62/PLOSOne_formatting_sample_title_authors_affiliations.pdf*](https://protect-eu.mimecast.com/s/V3pKCl714TowG6F17OKX?domain=journals.plos.org)

This has been done

*Please include additional information regarding the survey or questionnaire used in the study and ensure that you have provided sufficient details that others could replicate the analyses. For instance, if you developed a questionnaire as part of this study and it is not under a copyright more restrictive than CC-BY, please include a copy, in both the original language and English, as Supporting Information. In addition, please provide any details of pretesting of the questionnaire, if such pretesting took place, including the number of participants and where they were recruited from.*

The questionnaires developed for this study will be deposited with London School of Hygiene and Tropical Medicine’s (LSHTM) Data Compass and a link provided so anyone interested can have unrestricted access.

The questionnaire was pretested at the municipal hospital among adults aged 18 years or more routinely identified by a health worker as requiring a sputum TB test. A total of eight participants were recruited, 4 (50%) males and 4 (50%) females aged between 27-85 years (mean age: 55 year [SD:18]).

We have added two lines on pretesting of the questionnaire in the manuscript (line 117-120 on page 5) which read:

The questionnaire was pretested at the municipal hospital in a population similar to the study population. Following pretesting, revisions were made to improve comprehensibility and understanding.

*We note that you have stated that you will provide repository information for your data at acceptance. Should your manuscript be accepted for publication, we will hold it until you provide the relevant accession numbers or DOIs necessary to access your data. If you wish to make changes to your Data Availability statement, please describe these changes in your cover letter and we will update your Data Availability statement to reflect the information you provide.*

We wish to maintain that data will be deposited with the London School of Hygiene and Tropical Medicine’s Data Compass. However, due to the presence of sensitive patient information in the dataset, access will be restricted. It is recommended that persons who wish to access the dataset should click on the DOI link and then click on the “request access” button to fill a form which will be automatically submitted to the research data manager at London School of Hygiene and Tropical Medicine and to the first author. However, a non-author email address for request of the dataset is [researchdatamanagement@lshtm.ac.uk](mailto:researchdatamanagement@lshtm.ac.uk).

Thank you for reviewing our manuscript. We hope it now meets the criteria for publication. We look forward to hearing from you.

Yours sincerely

Joyce B. Der

Corresponding author
